# An Environmentally Friendly Compact Microfluidic Hydrodynamic Sequential Injection System Using *Curcuma putii* Maknoi & Jenjitt. Extract as a Natural Reagent for Colorimetric Determination of Total Iron in Water Samples

**DOI:** 10.1155/2023/3400863

**Published:** 2023-01-13

**Authors:** Maneerat Namjan, Natcha Kaewwonglom, Chonlada Dechakiatkrai Theerakarunwong, Jaroon Jakmunee, Wanpen Khongpet

**Affiliations:** ^1^Program of Chemistry, Faculty of Science and Technology, Nakhon Sawan Rajabhat University, Nakhon Sawan 60000, Thailand; ^2^Laboratory for Analytical Instrumentation and Electrochemistry Innovation, Department of Chemistry, Faculty of Science, Chiang Mai University, Chiang Mai 50200, Thailand; ^3^Environmental Science Research Center,and Center of Excellence for Innovation in Chemistry, Faculty of Science, Chiang Mai University, Chiang Mai 50200, Thailand

## Abstract

The miniaturization of analytical systems and the utilization of nontoxic natural extract from plants play significant roles for green analytical chemistry methodology. In this work, the microfluidic hydrodynamic sequential injection (HSI) with the LED-phototransistor colorimetric detection system has been proposed to create an ecofriendly and low-cost miniaturized analytical system for online determination of iron in water samples using *Curcuma putii* Maknoi & Jenjitt. extracts as high stability and good selectivity of a natural reagent. The proposed method was designed for online solution mixing and colorimetric detection on a microfluidic platform. The *Curcuma putii* Maknoi & Jenjitt. extracts and standard/samples were sequentially aspirated to fill the channel before entering the built-in flow cell. The intensity of iron-*Curcuma putii* Maknoi & Jenjitt. extract complex was monitored under the optimum conditions of flow rate, sample volume, mixing zone length, and aspiration sequences, by altering the gain control of the colorimetric detector to achieve good sensitivity. The results demonstrated a good performance of the green analytical systems. A linear calibration graph in the range of 0.5–6.0 mg L^−1^ was obtained with a limit of detection at an adequate level of 0.11 mg L^−1^ for water samples with a sample throughput of 30 h^−1^. The precise and accurate measurement results were achieved with relative standard deviations in the range of 1.61–1.72%, and percent recoveries were found in the range of 90.6–113.4. The proposed method offers cost-effective, easy operation over an appropriate analysis time (2 min/injection) with good sensitivity and is environmentally friendly with low consumption of solutions and the use of high stability and good selectivity of nontoxic reagents. The achieved method was demonstrated to be a good choice for routine analysis.

## 1. Introduction

Iron is one of the most important metals in the environmental geochemical, industrial, and biological processes. It can be chemically combined with many other elements to form iron ores. The natural deposits and refining of iron ores and industrial wastes are essential parameters for the existence of iron in natural water such as rivers, lakes, and groundwater. The iron level in groundwater can be increased by the dissolution of iron compounds in soils or ferrous boreholes and handpump components through leaching from home water drinking, cooking, washing, cleaning, and agricultural purposes [1, 2]. Generally, iron exists in natural fresh waters in the range of 0.5 to 50 mg L^−1^. The presence of iron above the certain level produces a reddish color of water, metallic taste, a distinct odor, turbidity, and a tendency to stain clothes. In submerged paddy fields, a ferric ion is reduced to the more soluble ferrous ion under the conditions of low pH and anaerobic environment. The excess ferrous form can lead to cytotoxicity in rice as it is absorbed by the roots and accumulated in plant tissues, leading to tissue destruction and yield loss [3, 4]. Therefore, monitoring of the iron levels in water is extremely essential for water quality evaluation and water pollution control to determine the suitability of water for consumption and other applications. The recommended limit of iron in groundwater and drinking water by the World Health Organization (WHO) is 0.3 mg L^−1^. Water for irrigation the iron levels above 0.1 mg L^−1^ may cause drip emitter clogging irrigation [5, 6].

Various analytical techniques have been developed for the determination of iron in water samples. These methods include ultraviolet-visible spectrophotometry (UV-Vis) [7, 8], atomic absorption spectroscopy (AAS) [9], liquid chromatography [10], potentiometry [11], and voltammetry [12]. Among these techniques, UV-Vis spectrophotometry is the analysis instrument with easy operation and simple colorimetric detection in the visible region with several reagents to form color complexes and can be encountered in almost every laboratory. However, this technique involves high cost and large sizes that consume high amounts of chemicals. Moreover, complete chemical reactions are important for the detection of the products. Thus, microfluidic technology is currently of interest for the development of a detection system with portability advantages, low-cost integrated miniaturized devices, providing a low solution consumption. To obtain automatic operation and sufficient sensitivity of the detection device, flow-based analytical systems such as flow injection (FI), sequential injection (SI), and hydrodynamic sequential injection (HSI) have gained more attention recently for good alternative choices. Among these flow-based techniques, HSI presents an excellent system in terms of cost, chemical consumption, and automatic operation. However, its drawbacks revealed to human health and environmental concerns because most of the reagents are toxic such as Tiron, 1,10 phenanthroline, 2-(5-bromo-2-pyridylazo)-5-[N-n-propyl-N-(3-sulfopropyl) amino] aniline, ferrozine, nitroso-R, 4,7-diphenyl-1,10-phenanthroline, bathophenanthroline, 2,2′-bipyridine, deferiprone, thiocyanate, and 3-hydroxy-1,2-dimethyl-4(1H)-pyridinone [2, 13–15]. To solve this problem, a natural reagent along with a microfluidic HSI system for the determination of iron is considered as a remarkable technique. The use of natural extract offers safer and greener colorimetric reagents for humans and the ecosystem. Some plant extracts were utilized as natural reagents in conjunction with digital images by a mobile phone and FI system for iron determination such as green tea [16], guava leaves [17], and *Phyllanthus emblica* Linn. [18] that showed successful quantitative analysis in real samples. In all of these plant extracts, phenolic compounds were found as common active compounds. They are comprised of a hydroxyl group (-OH) bonded directly to an aromatic ring and are capable of binding or chelating with metal ions. Thus, plant extracts containing phenolic compounds can be used as a natural complexing agent for metal ions.

*Curcuma putii* Maknoi & Jenjitt. is a new species discovered in central Thailand [19]. The leaves of this species are rich in phenolic compounds that have antioxidant activity. These compounds contain a large number of OH groups and a strong ability to complex with metals. Consequently, it is possible that *Curcuma putii* Maknoi & Jenjitt. extract could be applied as a great complexing agent with high stability, good selectivity, and sensitivity for iron quantification by the microfluidic HSI system.

Therefore, in this work, the combination of a compact and cost-effective microfluidic HSI and *Curcuma putii* Maknoi & Jenjitt. extract was proposed for the determination of total iron in water samples based on a green chemical analysis process. The proposed method exhibited higher stability than the other plant extracts that have previously been reported for iron determination and offered good sensitivity with a homemade colorimetric detection device. The special feature of the detector is its ability to adjust its sensitivity using a gain-adjustable signal. Typically, there is no distinction in the narrow concentration range since the generated signals all have the same shade. In this work, amplification of the signal with adjustable gain can assist to distinguish a small concentration range. The satisfactorily selectivity and reproducibility results were achieved with a portable device and an environmentally friendly approach which is suitable for water quality monitoring.

## 2. Materials and Methods

### 2.1. Chemicals and Materials

All chemicals were of analytical reagent grade, and deionized water was used for the preparation of all solutions throughout the experiment. A stock standard solution of iron (II) at 1000 mg L^−1^ was prepared by dissolving 0.7022 g of ammonium ferrous sulfate hexahydrate (Sigma-Aldrich, England) in water containing 1.0% (v/v) concentrated sulfuric acid (BDH, England); then, the volume was adjusted to 100 mL. The stock standard solution of iron (III) at 1000 mg L^−1^ was prepared in the same way with 0.1 g of ferric chloride (BDH, England). A series of working standard solutions were prepared daily by diluting the stock standard solution to the desired concentrations. Acetate buffer (0.2 M, 500 mL) pH 4.8 was prepared by dissolving 7.21 g of sodium acetate trihydrate (Carlo Erba, Italy) in water containing 2.72 mL of acetic acid (Carlo Erba, Italy).

### 2.2. *Curcuma putii* Maknoi & Jenjitt. Extraction

Dry *Curcuma putii* Maknoi & Jenjitt. leaves of 1.0 g were extracted with 40 mL of 70% (v/v) ethanol using ultrasonic for 1 hour and subsequently filtered through a filter paper (Whatman No. 4). For ongoing usage during the day, the filtrate was maintained at room temperature. The extract was prepared from fresh *Curcuma putii* Maknoi & Jenjitt. leaves daily, and the concentration of the total phenolic component was also determined before use. The use of the same extraction methods with the same batch of *Curcuma putii* Maknoi & Jenjitt. leaves was one of these criteria. The total phenolic contents of *Curcuma putii* Maknoi & Jenjitt. extracts were quantified using the Folin–Ciocalteau assay [20] and were found to be 2,021 mg L^−1^ (80.33 ± 2.12 mg/g dry weight), which was employed in the experiment.

The most active compounds of phenolic compounds, including flavonoids and tannins, have the ability to chelate metal ions. This research believed that flavonoids or tannins were the major compounds in *Curcuma putii* Maknoi & Jenjitt. extract for chelating with metal. Therefore, the ferric test and Shinoda's test procedures were used for tannins and flavonoids determination, respectively [21, 22]. Moreover, tannins are generally classified into hydrolysable (gallotannins and ellagitannins), condensed, and complex tannins. Therefore, the analysis of tannins in *Curcuma putii* Maknoi & Jenjitt. was preliminarily studied using the acid butanol test, nitrous acid test [21], and potassium iodate test [23]. The acid butanol test is specific for condensed tannins, forming a red-orange to red-crimson product. Hydrolysable tannins ellagitannins can be determined with a nitrous acid test, which forming a red or pink color and slowly changing color to purple or blue while potassium iodate can also be used for the detection of gallotannins, forming pink color.

To confirm the major active compound in *Curcuma putii* Maknoi & Jenjitt. extract, liquid chromatography-mass spectrometry (LC-MS) was used for the identification of phenolic compounds. This method mainly aims to identify the compounds present in the extracts. LC-MS was used under the following instrumental operating conditions: column-Hypersil GOLDTM PFP (100 × 2.1 mm i.d., 1.9 *μ*m), mobile phase: (*A*) 0.1% formic acid in water and (*B*) methanol, gradient elution: 0% B in A for 2 min, linear increasing from 0% *B* in *A* to 100% *B* in *A* for 8 min, and 100% *B* for 2 min, flow rate 1 mL/min, and detection: UV 254 nm.

### 2.3. Study of the *Curcuma putii* Maknoi & Jenjitt. Extract-Iron Complex Formation

In this study, 10 and 100 mg L^−1^ of both iron (II) and iron (III) complexes with *Curcuma putii* Maknoi & Jenjitt. extracts were extracted by using various solvents including deionized water, acetate buffer (pH 4.8), and ethanol in order to study the extraction efficiency. The procedure is briefly described as follows: Both 2.5 mL standard solutions of iron (II) and iron (III) of 1,000 mg L^−1^ were mixed with 10 mL of *Curcuma putii* Maknoi & Jenjitt. extract in three extraction solvents (water, acetate buffer pH 4.8, and ethanol) and adjusted to the final volume of 25 mL with acetate buffer. After 30 min, the mixtures of each extraction solvent were scanned for absorption spectra measurement at the visible range of 500–800 nm using a UV-Vis spectrophotometer (Thermo Fisher Scientific, USA). The *Curcuma putii* Maknoi & Jenjitt. extracted without iron was used as a blank solution. The complex formation of both 10 mg L^−1^ iron (II) and iron (III) with *Curcuma putii* Maknoi & Jenjitt. extracts were prepared similarly but with 100 mg L^−1^ standard solutions of iron (II) and iron (III).

### 2.4. Stability Study of the *Curcuma putii* Maknoi & Jenjitt. Extract

The *Curcuma putii* Maknoi & Jenjitt. extract was prepared in the suitable extract solvent from the previous experiment and let to stand in the brown bottle at room temperature for 0–7 hours and up to 30 days before mixing with 10 mg L^−1^ of iron (III). The mixtures were prepared as previously described. Finally, the absorption spectra were measured to investigate absorption characteristics.

### 2.5. Microfluidic HSI Design and Assembly

**Figure 1:**
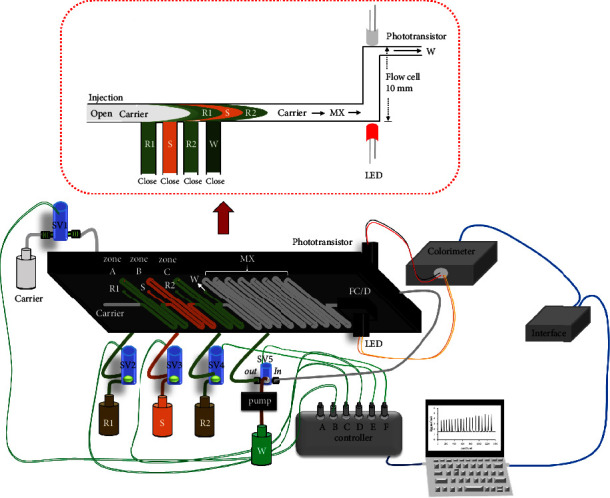
The schematic of the microfluidic HSI system for determination of total iron, using acetate buffer solution as a carrier, where *S* = standard/sample, *R*1 and *R*2 = natural reagent, MX = mixing zone, FC = flow cell, *D* = homemade LED/phototransistor colorimeter, SV = solenoid valve, and *W* = waste.

The microfluidic HSI colorimetric system (Figure 1) was designed for the determination of total iron using *Curcuma putii* Maknoi & Jenjitt. extract as a natural reagent. It consisted of a 5.0 mm thickness black polymethyl methacrylate (PMMA) platform (80 mm × 140 mm) that contained a microfluidic channel (0.5 mm width × 0.5 mm deep) and a built-in flow cell with a laser cutting machine (MK2230, Mass Business Co. Ltd., Thailand) for online sampling and reagents mixing and product detection, 1.0 mm thickness clear PMMA (80 mm × 140 mm) as a top cover on the platform, and peristaltic pump (Ismatec, Model ISM796B-230 V, Switzerland) fitted with Tygon pump tubings (1.14 mm i.d.) which were connected to PTFE tubing in each port of the microfluidic channel on the platform. All ports were connected with 2-way solenoid valves (Biochem Valve, 075T2NC12-32, 20 PSI 12 VDC, USA) or a 3-way solenoid valve (NResearch, HP161T031, 12 VDC 100 PSI, USA) which was controlled by Control Solenoid Valve 3.0 software of a homemade controller connected to the personal computer to detect the flow of solutions by turning on/off the valves, and a simple homemade light-emitting diode phototransistor (LED-PT)-based colorimetric detector using a red LED (maximum emission: 660 nm) as a light source which was assembled in a built-in flow cell in the microfluidic platform, and a data acquisition unit (eDAQ Australia).

### 2.6. Detection of Total Iron

First of all, the microfluidic HSI colorimetric system (Figure 1) was cleaned thoroughly with deionized water by switching solenoid valves (SV1–SV5) to the flow cell position for 2 minutes and propelling the carrier solution (acetate buffer) for 2 minutes. Then the *Curcuma putii* Maknoi & Jenjitt. extract was aspirated into the microfluidic channel on the platform for 6 seconds by a peristaltic pump, 2-way solenoid valve 2 (SV2), and 3-way solenoid valve 5 (SV5, position “out”) while 2-way solenoid valves 1, 3, and 4 (SV1, SV3, SV4) were closed. In this step, the solution was filled into the zone *A* (50 *μ*L, *R*1), and the excess volume was discarded into the reservoir for waste collection. The aspiration of the standard solution of iron (III)/sample solution (75 *μ*L zone *B*, *S*) and *Curcuma putii* Maknoi & Jenjitt. extract (50 *μ*L zone *C*, *R*2) were similarly operated as previously described, but 2-way solenoid valve 3 (SV3) and 2-way solenoid valve 4 (SV4) were opened for 8 and 6 seconds to fill in the zone *B* and zone *C*, respectively. Thus, the sequence of solution zones was *R*1 *S R*2, respectively, for efficient mixing between the natural reagent and standard/sample solution. Finally, injection step, all solution zones were pushed by acetate buffer carrier stream through the mixing zone with 2-way solenoid valve 1 (SV1) into the flow cell with 10 mm path length to monitor the color of complex formation and record the signal profiles continuously on a personal computer. Each new cycle was continuously operated with the control solenoid valve 3.0 software of a homemade controller. In addition, the solution aspiration was controlled by opening only one 2-way solenoid valve per step and switching 3-way solenoid valve 5 at the position “out” while switching 3-way solenoid valve 5 at the position “in” for colorimetric detection of *Curcuma putii* Maknoi & Jenjitt. extract-iron complex formation.

### 2.7. Sample Preparation

Water samples were collected of different sources including well water samples and tap water from Sakae Krang Sub-District, Mueang District, Uthai Thani, Thailand. Samples were filtered through a Whatman filter paper No. 6 and kept in polyethylene bottles at 4°C. All bottles were rinsed with 10% (v/v) of nitric acid or sulfuric acid and triple-rinsed with distilled water before further usage to reduce metal ion sorption from the sample storage bottle. The samples were analyzed within 24 hours after sampling.

To investigate the efficient detection of the proposed method for the determination of total iron, the standard samples were prepared using the known concentration of the iron standard solution to give three standard samples that contained iron (II), iron (III), and iron (II) plus iron (III). Each sample has the final concentration of 2.0 mg L^−1^ as a reference value.

## 3. Results and Discussion

### 3.1. Preliminary Study

#### 3.1.1. Identification of Phenolic Compounds in *Curcuma putii* Maknoi & Jenjitt

Identification of phenolic compounds was performed according to screening methods of the Shinoda's test, ferric test, acid butanol test, nitrous acid test, and potassium iodate test. The results are shown in Table 1. In the test for flavonoids with Shinoda's test procedure, the result was found that the formation of brown color appear that indicated the presence of flavonoids. Tannins detection with ferric test procedure was tested with 2.0% (w/v) of ferric chloride, resulting in a greenish-brown precipitates appearing in the extract. It is possible that the phenolics detected were tannins. Some research reported that the ferric test could be used to distinguish hydrolysable tannins from condensed tannins. Condensed tannins give greenish-brown precipitates while hydrolysable tannins form bluish-black color and precipitates [21]. Therefore, it is possible that the extracts contain condensed tannins. To confirm that *Curcuma putii* Maknoi & Jenjitt. extract contains condensed tannins (e.g., catechin and gallocatechin), the extract was tested using acid butanol, nitrous acid, and potassium iodate. The formation of a crimson product with the acid butanol test indicates the presence of condensed tannins in *Curcuma putii* Maknoi & Jenjitt. while hydrolysable tannins (ellagitannins and gallotannins) with nitrous acid and potassium iodate tests, gave negative results. Therefore, the preliminary conclusion is that the phenolic compounds in *Curcuma putii* Maknoi & Jenjitt. including flavonoids and condensed tannins were obtained.

**Figure 2:**
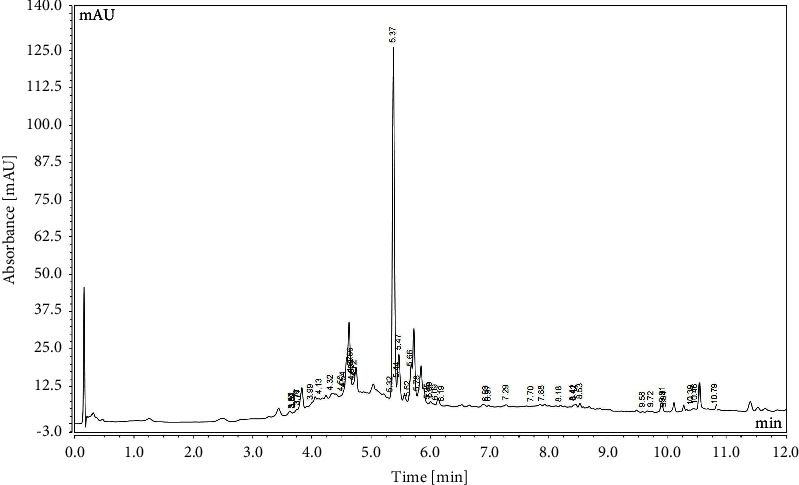
Chromatogram of the *Curcuma putii* Maknoi & Jenjitt. extract.

**Figure 3:**
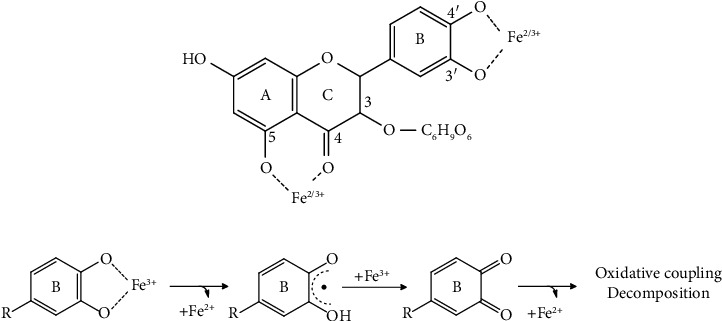
The proposed complexation mechanism of quercetin 3-O-glucuronide and iron. (a) Possible chelating sites of iron (iron (III) or iron (II)) ions on quercetin 3-O-glucuronide. (b) Oxidation of the flavonoid 3ʹ,4ʹ-dihydroxyl (catechol group) site to a semiquinone-type radical and possibly to a quinone [25].

The species of phenolic compounds in *Curcuma putii* Maknoi & Jenjitt. was confirmed by LC-MS. The results are shown in Table 2 and Figure 2. The *Curcuma putii* Maknoi & Jenjitt. extract contains flavonoids, condensed tannins, and other compounds consistent with the preliminary results of screening methods. The major compounds of *Curcuma putii* Maknoi & Jenjitt. extract were miquelianin (quercetin 3-O-glucuronide) with the retention time of 5.37 which is a species of natural flavonoid and metabolite of quercetin. This compound has the carbonyl group in the C ring and multiple hydroxyl groups and has several chelating sites for the complexation of metals. The possible chelating site of iron consists of the C4-carbonyl-C5-hydroxy and the ortho-dihydroxyl (3′,4′-dihydroxyl or catechol) groups [24]. Therefore, it is possible that quercetin 3-O-glucuronide is the main active compound in *Curcuma putii* Maknoi & Jenjitt. for iron chelation. The proposed mechanisms of iron reactions with quercetin 3-O-glucuronide is shown in Figures 3(a) and 3(b). Quercetin is a strong chelating agent with both iron (II) and iron (III). Iron attached to the 4-carbonyl group of C ring and 5-hydroxyl group of A ring, resulting in the break of double bond and deprotonation to generate the two Fe-O bonds. The next possible site is the 3′,4′-dihydroxyl site, either one or both *H* atoms can be removed from hydroxyl groups and bonded with iron. Moreover, iron (II) and iron (III) ions are most likely chelated with two quercetin molecules in 1 : 2 and 1 : 1 ratios, respectively. However, the formation of iron-quercetin or metal-ligand complex depends on pH, solvent, oxidizing agent, and reactant forms [24, 26].

**Figure 4:**
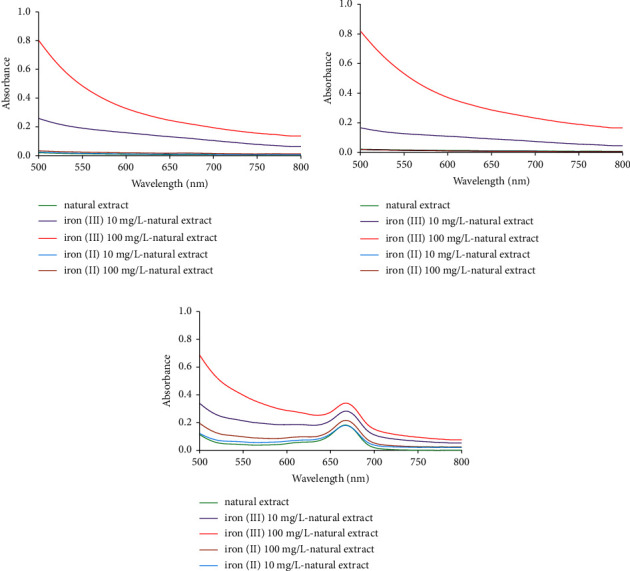
(a) The absorbance spectra of the *Curcuma putii* Maknoi & Jenjitt. extract-iron complex in water. (b) The absorbance spectra of the *Curcuma putii* Maknoi & Jenjitt. extract-iron complex in acetate buffer solution. (c) The absorbance spectra of the *Curcuma putii* Maknoi & Jenjitt. extract-iron complex in ethanol.

Several studies have reported that quercetin has a stronger affinity for iron (III) than for iron (II) [27–29]. Furthermore, iron (III) at pH 5.0 could oxidize sufficiently to be reduced to iron (II) by quercetin (Figure 4(b)) [29]. Bijlsma et al. reported the reduction of iron (III) to iron (II) through the transfer of electrons from the flavonoid (catechol group) to iron and the flavonoid is oxidized to a semiquinone radical, and finally to a quinone [25].

#### 3.1.2. *Curcuma putii* Maknoi & Jenjitt. Extract-Iron Complex Formation

The absorption spectra of iron (III), iron (II), and *Curcuma putii* Maknoi & Jenjitt. extract in water, acetate buffer, and ethanol were recorded using UV-Vis spectrophotometer (Thermo Fisher Scientific, USA) in the range of 500–800 nm. Figures 4(a)–4(c) represent the absorption spectra of *Curcuma putii* Maknoi & Jenjitt. extract-iron complexes in water, acetate buffer, and ethanol, respectively. The results indicated that the use of water and acetate buffer as the extraction solvents (Figures 4(a) and 4(b)) might not be able to extract the active species from the *Curcuma putii* Maknoi & Jenjitt., which was the main active component for chelating with iron. It could also be possible that the formation of iron and the active site of the active species did not appear, resulting in no significant detection of iron complex absorption in the 650–700 nm region, and their absorption spectra were similar to those of iron standard solution prepared in water and acetate buffer. In ethanol, both iron (II) and iron (III) could bind to the active site of the active species of *Curcuma putii* Maknoi & Jenjitt. a blue-green or brown color appears and exhibits the maximum absorption at 667 nm as shown in Figure 4(c). However, the absorbances values of iron (III) complexes were higher than those of iron (II) complexes. This is probably due to the fact that the iron (III)-*Curcuma putii* Maknoi & Jenjitt. complex has a higher molar absorptivity and stronger affinity. Thus, iron (III) standard solution was chosen for the construction of calibration curves for further studies and determination of total iron by the microfluidic HSI colorimetric system in order to obtain the best sensitivity for water sample monitoring by using the microfluidic system.

**Figure 5:**
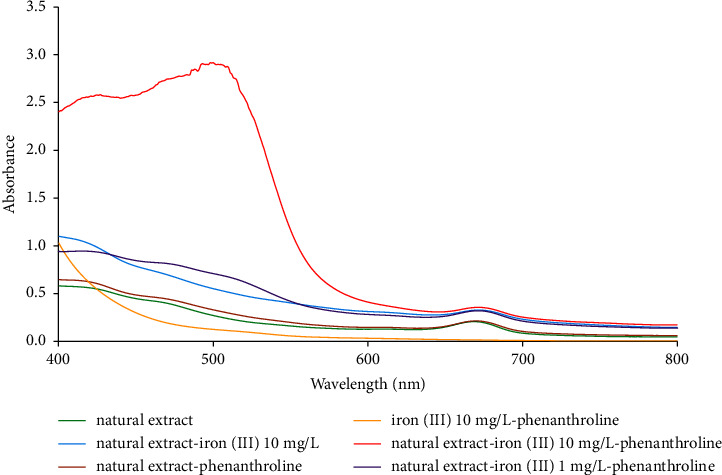
The absorbance spectra studies for the reducing effect of *Curcuma putii* Maknoi & Jenjitt. extract (natural extract).

Some research studies reported that some chemicals (e.g., flavonoids and tannins) could reduce iron (III) to iron (II). Therefore, to test for the reducing effect of the sample, preliminary studies were conducted to investigate some chemicals (e.g., flavonoid, tannin, steroid, and terpenoids.) found in the *Curcuma putii* Maknoi & Jenjitt. extract. From the report based on the phenanthroline method, iron (III) did not form a reddish complex with phenanthroline but only with iron (II) as it appeared to show the absorption spectra at about 510 nm [30] or 500 ± 20 nm [17]. Thus, if the active species in *Curcuma putii* Maknoi & Jenjitt. extract could reduce iron (III) to iron (II), a reddish complex of iron (II) and phenanthroline would be formed, leading to the absorption spectra at about 500 ± 20 nm. In the experiment, iron (III) was mixed with *Curcuma putii* Maknoi & Jenjitt. extract and 3.0% (w/v) phenanthroline. The mixing procedure is similar to that in Section 2.3 but phenanthroline was added before adjusting the final volume. In addition, the desired final concentrations were 1.0 and 10 mg L^−1^ of iron (III) and 3.0% (w/v) of phenanthroline. This mixed solution was scanned across the regions of 400–800 nm to compare with other various groups such as iron (III)-*Curcuma putii* Maknoi & Jenjitt. extract, iron (III)-phenanthroline, *Curcuma putii* Maknoi & Jenjitt. extract-phenanthroline, and *Curcuma putii* Maknoi & Jenjitt. extract. The results indicated that *Curcuma putii* Maknoi & Jenjitt. extract has a natural reducing effect (Figure 5). The addition of phenanthroline into the solution that contained *Curcuma putii* Maknoi & Jenjitt. extract and iron (III) clearly caused a reddish color, resulting in an appearance of absorption spectra at about 502 nm wavelength.

#### 3.1.3. Stability of the *Curcuma putii* Maknoi & Jenjitt. Extract

**Figure 6:**
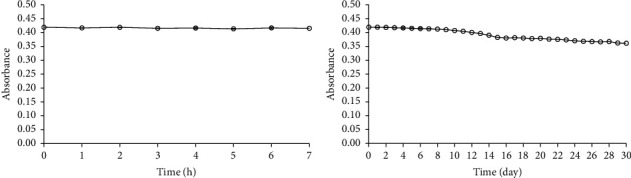
Stability of *Curcuma putii* Maknoi & Jenjitt. extract. (a) 0–7 hours. (b) 0–30 days before mixing with 10 mg L^−1^ of iron (III) solutions.

*Curcuma putii* Maknoi & Jenjitt. extract was prepared and left for 0–7 hours and up to 30 days before being mixed with 10 mg L^−1^ of iron (III). The absorbances values are recorded as shown in Figures 6(a) and 6(b). The deviation between the initial signal (first measurement point) to the extract signal after 20 days of storage was 3.93% The percentage difference of signal between the initial signal (first measurement point) and the extract storage signal for 20 days was 9.55%, which is calculated by dividing the difference between the target signal (storage 20 days) and the initial signal by the initial signal and multiplying the result by 100, both did not exceed 10%. For more than 20 days, the results provided 5.23% for the deviation between the initial signal to the final signal (stored for 30 days) and 13.84% for the percentage difference between the initial signal and the final signal (stored for 30 days), respectively. The results indicated that *Curcuma putii* Maknoi & Jenjitt. extract has a high stability of the determination of total iron, while other plant extracts whose stability has been reported at least within 4 and 72 hours (3 days) [16, 17].

### 3.2. Optimization

The microfluidic HSI colorimetry was used for determination of total iron by varying important parameters of experimental conditions, such as flow rate, sample volume, mixing zone length, and aspiration sequences. The investigated parameter was varied while the others were fixed. The iron (III) standard solutions of 0.0–6.0 mg L^−1^ were employed during optimization. Then, the optimum conditions were selected to obtain high sensitivity and sufficiency for total iron determination.

#### 3.2.1. Effect of Flow Rate

**Figure 7:**
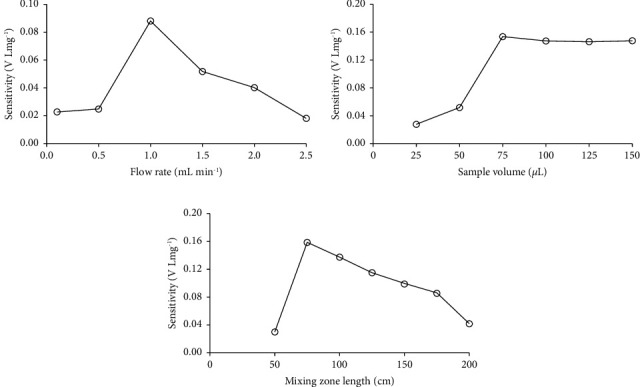
Effect of (a) flow rate, (b) sample volume, and (c) mixing zone length on sensitivity of iron (III) determination.

Flow rate was the one of influencing parameters on the sensitivity of *Curcuma putii* Maknoi & Jenjitt. extract-iron complex detection because it affected solution dispersion in a microfluidic platform. The results shown in Figure 7(a) indicate that a low flow rate led to a longer time for both solution aspiration and the movement into the detector, resulting in a large dispersion which could reduce the sensitivity and the sample throughput. The outcome of increasing flow rate from 0.1 to 1.0 mL min^−1^ was a decrease in dispersion thus causing higher sensitivity. However, at flow rate above 1.0 mL min^−1^, a lower sensitivity was obtained due to the limited reaction time. So, a flow rate of 1.0 mL min^−1^ was selected.

#### 3.2.2. Effect of Sample Volume

The sample volumes were controlled with the microfluidic channel on the platform which was designed in accordance with calculating length, width, and depth of the *S* zone position to obtain volume in the range of 25–150 *μ*L, as shown in Figure 7(b). The sensitivity increased with the increase in sample volume up to 75 *μ*L, then remained constant. Thus, to achieve high sensitivity and sampling rate, a sample volume of 75 *μ*L was selected for further studies.

#### 3.2.3. Effect of Mixing Zone Length

In order to achieve an efficient mixing of sample and reagent, the carrier was required for the flow cell throughput, leading to maximum sensitivity for product detection. The mixing zone lengths of 50–200 cm were studied. The results shown in Figure 7(c) also indicate that the sensitivity increased with the increase of mixing zone length up to 75 cm and rapidly decrease from 75 to 200 cm because increasing the length of the mixing zone led to extremely large dispersion of the detection zone, resulting in lower sensitivity. Thus, a 75 cm mixing zone length was chosen because it provided the highest sensitivity.

#### 3.2.4. Effect of Aspiration Sequence

The aspiration sequences of the solution are studied by using four patterns with different carrier solutions as shown in Table 3. The results revealed that the pattern of reagent (zone *R*), sample (zone *S*), and reagent (zone *R*), using a natural reagent as a carrier provided the highest sensitivity. However, some drawbacks often occurred during the experiment operation such as the color of the natural reagent easily and quickly adhered to the microfluidic channel. This caused small colloids that were retained in the flow cell, resulting in an unstable signal, unreliable, and faulty results. To compromise between sensitivity and reduce these drawbacks, the sequence of reagent (*R*), sample (*S*), and reagent (*R*) with acetate buffer as a carrier (No. 2) was selected.

### 3.3. Analytical Characteristics of the Microfluidic HSI Colorimetric System

**Figure 8:**
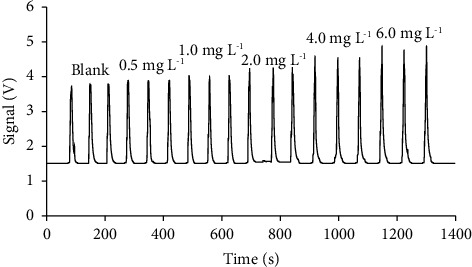
Response profile for the determination of iron (III) using the microfluidic HSI colorimetric system.

The analytical characteristics of the microfluidic HSI colorimetric system using *Curcuma putii* Maknoi & Jenjitt. extract as a natural reagent include linearity ranges, the limit of detection (LOD), the precision, and the accuracy. The selected conditions were obtained as previously studied. A linear calibration graph in the range of 0.5–6.0 mg L^−1^ (*y* = 0.1586*x* − 0.0109, *r*^2^ = 0.9992) was constructed with a sample throughput of 30 h^−1^ and approximately 1.7 mL/operation of waste as shown in Figure 8. The limit of detection was achieved at 0.11 mg L^−1^, which was calculated by dividing three times standard deviation of the intercept by the slope of the calibration graph. The precision was evaluated by the aspiration of iron (III) standard solutions of 0.5, 2.0, and 6.0 mg L^−1^ for 11 replicates, that obtained relative standard deviation percentages (%RSD) of 1.72, 1.63, and 1.61, respectively, resulting in high precision of the measurements. The percentages of recovery were studied by spiking the diluted water samples and the standard samples with known different concentrations of iron (III) solution (1.0 and 3.0 mg L^−1^). The results are summarized in Table 4, indicating that the percentage of recovery was in the range of 90.6–113.4 with satisfactory accuracy and correspondence with the AOAC acceptable range.

### 3.4. Interference Study

The effects of some interference species on iron determination were studied, especially cations and anions such as Zn^2+^, Cu^2+^, Mn^2+^, Cd^2+^, Ni^2+^, Cr^3+^, Co^2+^, Pb^2+^, Na^+^, Ca^2+^, Mg^2+^, Al^3+^, NH_4_^+^, Cl^−^, NO_2_^−^, NO_3_^−^, and PO_4_^3−^. These species of known concentration were added to iron (III) standard solution containing a fixed concentration of 1.0 mg L^−1^ to evaluate the tolerance limit of interference species on iron detection. The tolerance limit was defined as the maximum of interference species concentration that caused the iron concentration deviation of less than ±5% of the iron concentration without interference. It was found that there was no interference in excess of 500 mg L^−1^ of Na^+^, Ca^2+^, and Cl^−^, 100 mg L^−1^ of Al^3+^, 50 mg L^−1^ of Mg^2+^ and PO_4_^3−^, 25 mg L^−1^ of NO_3_^−^, 10 mg L^−1^ of NH_4_^+^, 5 mg L^−1^ of Cu^2+^, 1.0 mg L^−1^ of Mn^2+^, Zn^2+^, Ni^2+^, Cd^2+^, and Pb^2+^, indicating that some species (Mn^2+^, Zn^2+^, Ni^2+^, Cd^2+^, and Pb^2+^) had a positive or negative effect that interfered the determination of total iron. However, these species were absent in the studied samples. Thus, it could be considered to have no interference effect in this case.

### 3.5. Application to Real Samples

The microfluidic HSI system combining a natural reagent from *Curcuma putii* Maknoi & Jenjitt. extract (new plant species) was applied to determine iron ion in water samples. Comparing the spectrophotometric method that used 1,10 phenanthroline as a chromogenic reagent and hydroxylamine as a reducing agent, the results as shown in Table 5 indicate that the proposed microfluidic HSI-*Curcuma putii* Maknoi & Jenjitt. extract system had good correlation with the spectrophotometric method. Considering the linear regression equations (*y* = 0.9779*x* + 0.0824) of the correlation graph between both methods, where *X* and *Y* represented the results obtained from spectrophotometric and the microfluidic HSI methods, the slope was close to 1 and *r*^2^ value was 0.9918. Moreover, both methods were also compared using the *t*-test at 95% confident level [31]. It was found that there was no significant difference among all water samples (*t*_critical_ = 2.36, *t*_calculated_ = 0.16, DOF = 7). Moreover, when comparing the amount of total iron obtained from standard samples (sample numbers 6–8) with the amount of total iron (2.0 mg L^−1^) obtained from iron (II) and iron (III) standard solutions, it was found that there was no significant difference. All results indicated that the proposed microfluidic HSI-*Curcuma putii* Maknoi & Jenjitt. extract system gave a comparable performance to the spectrophotometric method. Moreover, the proposed method could reduce some major drawbacks of the standard spectrometric method. The first point, the proposed method successfully determined total iron in water samples with microliter-volumes of nontoxic reagents and samples, while the standard spectrometric method required volume of milliliter of highly toxic reagents. It indicated that the proposed method produced less toxic waste and was more environmentally friendly. The second avail, the detection device of the proposed method is much cheaper than a standard spectrometric instrument and could be easily fabricated. Besides, the automatic operation of the detection system could shorten the analysis time, which is convenient for routine work. However, the proposed method has a limitation from the air bubble within the solutions that could interfere with the detection signal of the microfluidic HSI system. This drawback could be solved by removing the air bubble in the microfluidic HSI with the carrier solution during the operation and degassing the solutions before use.

## 4. Conclusions

A compact microfluidic HSI system for homemade colorimetric detection using *Curcuma putii* Maknoi & Jenjitt. extract was proposed as a green analytical methodology for iron quantification. The extract was nontoxic, low in cost, had high stability (stable for at least 20 days) with good selectivity, and was an easily available reagent that could be applied to the microfluidic HSI system. The developed method offered high accuracy and precision as well as sufficient sensitivity with the adjustable gain control detection device and optimum conditions without the need to purify natural reagents prior to use. The proposed method provided cost-effective, easy operation with an appropriate analysis time (2 min/injection) and environmental protection with low consumption of solutions and chemical usage reduction. Therefore, it could be considered as an alternative analytical method for routine analysis of total iron in water samples.

## Figures and Tables

**Table 1:** Colorimetric test results.

Methods	Positive/Negative	Colour observed	
Ferric test	+	Greenish-brown	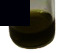
Shinoda's test	+	Brown	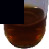
Acid butanol test	+	Crimson	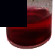
Nitrous acid test	−	Brownish-yellow	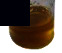
Potassium iodate test	−	Brown	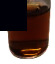

+ positive test, − negative test.

**Table 2:** Characterization of compounds from *Curcuma putii* Maknoi & Jenjitt. extract by the LC-MS analysis.

No	Proposed compounds	Retention time	Formula	Molecular	m/z
				Weight	
1	1,5-anhydro-1-[5,7-dihydroxy-2-(4-hydroxyphenyl)-4-oxo-4H-chromen-8-yl]-2-O-[(2*E*)-3-(4-hydroxyphenyl)-2-propenoyl] hexitol	3.67	C_30_H_26_O_12_	578.1420	577.1346
2	Catechin	3.69	C_15_H_14_O_6_	290.0787	289.0715
3	*p*-coumaric acid glucoside	3.76	C_15_H_18_O_8_	326.0999	325.0927
4	1-naphthol glucuronide	3.76	C_16_H_16_O_7_	320.0893	319.0820
5	(+)-gallocatechin	3.77	C_15_H_14_O_7_	306.0737	305.0664
6	Sinapinic acid-*O*-glucuronide isomer	3.99	C_17_H_20_O_11_	400.1004	399.0931
7	Heptaethylene glycol	4.13	C_14_H_30_O_8_	326.1940	349.1832
8	Catechin	4.32	C_15_H_14_O_6_	290.0789	289.0716
9	8-*O*-methylfusarubin	4.50	C_16_H_16_O_7_	320.0894	319.0820
10	*p*-coumaric acid glucoside	4.54	C_15_H_18_O_8_	326.0999	325.0927
11	3,5,6,7-Tetrahydroxy-2-(4-hydroxyphenyl)-2,3-dihydro-4H-chromen-4-one	4.66	C_15_H_12_O_7_	304.0583	303.0510
12	Leucyl-leucyl-norleucine	4.67	C_18_H_35_N_3_O_4_	357.2627	358.2700
13	Epiafzelechin	4.68	C_15_H_14_O_5_	274.0841	275.0913
14	Guibourtinidol-(4alpha->6)-catechin	4.72	C_30_H_26_O_10_	546.1523	545.1449
15	3,4-methylenesebacic acid	5.32	C_12_H_18_O_4_	226.1205	249.1097
16	Miquelianin	5.37	C_21_H_18_O_13_	478.0745	477.0671
17	Isoquercetin	5.44	C_21_H_20_O_12_	464.0954	463.0880
18	Rutin	5.47	C_27_H_30_O_16_	610.1527	609.1455
19	Bergaptol	5.62	C_11_H_6_O_4_	202.0265	201.0192
20	Trifolin	5.66	C_21_H_20_O_11_	448.1005	447.0931
21	Flaviolin	5.78	C_10_H_6_O_5_	206.0215	205.0143
22	7-hydroxy-9-methoxy-6-(1,3,4-trihydroxy-2-butanyl)-1,2-dihydrocyclopenta[c]chromene-3,4-dione	5.95	C_17_H_18_O_8_	350.0997	349.0925
23	Dodecyltrimethylammonium	5.99	C_15_H_33_N	227.2612	228.2685
24	Bis(2-ethylhexyl) amine	6.09	C_16_H_35_N	241.2766	242.2839
25	3-tert-butyladipic acid	6.19	C_10_H_18_O_4_	202.1206	201.1133
26	Prometryn	6.93	C_10_H_19_N_5_S	241.1363	242.1436
27	3-oxolauric acid	6.97	C_12_H_22_O_3_	214.1569	237.1461
28	trans-2-Dodecenoylcarnitine	7.29	C_19_H_35_NO_4_	341.2566	342.2639
29	*N*,*N*-Bis(2-hydroxyethyl)dodecanamide	7.70	C_16_H_33_NO_3_	287.2461	310.2354
30	3,5-di-tert-Butyl-4-hydroxybenzaldehyde	7.88	C_15_H_22_O_2_	234.1620	235.1693
31	10,16-dihydroxyhexadecanoic acid	8.41	C_19_H_38_N_2_O_3_	342.2882	343.2955
32	(Similar to: (1*S*,8*S*,9*S*,10*S*,13*R*)-6,9,10-trimethyl-2-oxo-4,14-dioxatetracyclo-tetradeca-3(7),5-dien-8-yl acetate; Δmass: 38.1571 Da)	8.42	C_15_H_29_NO	239.2249	240.2322
33	(Similar to: (1S,8S,9S,10S,13R)-6,9,10-trimethyl-2-oxo-4,14-dioxatetracyclo-tetradeca-3(7),5-dien-8-yl acetate; Δmass: −64.9062 Da)	8.53	C_20_H_26_O_4_	330.1831	329.1758
34	5,5′-diisopropyl-2,2′-dimethyl-3,3′,4,4′-biphenyltetrol	9.58	C_16_H_33_NO	255.2560	256.2633
35	Hexadecanamide	9.72	C_19_H_38_O_4_	330.2770	353.2662
36	L-*α*-PALMITIN	9.91	C_24_H_38_O_4_	390.2769	413.2661
37	Bis(2-ethylhexyl) phthalate	9.94	C_18_H_34_O_2_	282.2559	281.2486
38	Oleic acid	10.39	C_37_H_67_O_8_P	670.4573	669.4496
39	(2*R*)-1-(Palmitoyloxy)-3-(phosphonooxy)-2-propanyl (9*Z*,12*Z*,15*Z*)-9,12,15-octadecatrienoate	10.46	C_29_H_50_O_3_	446.3760	469.3651
40	13-hydroxy-alpha-tocopherol	10.79	C_29_H_48_O_3_	444.3591	477.3917

**Table 3:** Study on aspiration sequences.

No	Carrier solution	Sequence^*a*^	Calibration graph equation	*r* ^2^
1	deionized water	*R*/*S*/*R*	*y* = 0.0301*x* + 0.1368	0.9964
2	Acetate buffer	*R*/*S*/*R*	*y* = 0.1586*x* − 0.0109	0.9992
3	Natural reagent	*R*/*S*/*R*	*y* = 0.3253*x* − 0.2195	0.9746
4	Standard/sample solution	*S*/*R*/*S*	*y* = 0.1094*x* + 0.1678	0.9931

^*a*^ *R* and *S* represent natural reagent and standard/sample, respectively.

**Table 4:** Concentration of total iron in water samples and percent recoveries.

Sample	Iron added (mg L^−1^)	Iron found (mg L^−1^)	Recovery (%)
1	0	3.085	—
	1	4.151	106.6
	3	6.301	107.2

2	0	1.076	—
	1	2.111	103.5
	3	4.351	109.2

3	0	0.661	—
	1	1.751	109.0
	3	3.379	90.6

4	0	2.359	—
	1	3.398	103.9
	3	5.630	109.0

5	0	2.477	—
	1	3.446	96.9
	3	5.879	113.4

6	0	2.003	—
	1	2.976	97.3
	3	5.102	103.3

7	0	2.083	—
	1	3.108	102.5
	3	4.819	91.2

8	0	2.089	—
	1	3.037	94.8
	3	5.063	99.1

**Table 5:** Comparison of the proposed method and spectrophotometric method for the determination of total iron in water samples.

Sample	*Total iron (mg L* ^*−1*^)^*a*^	
	Proposed method	Spectrophotometric method
1	6.17 ± 0.08	6.94 ± 0.01
2	2.15 ± 0.01	1.93 ± 0.01
3	1.32 ± 0.13	1.11 ± 0.03
4	11.79 ± 0.05	12.08 ± 0.09
5	9.91 ± 0.08	9.40 ± 0.07
6	2.00 ± 0.13	1.94 ± 0.01
7	2.08 ± 0.03	2.11 ± 0.01
8	2.09 ± 0.06	2.19 ± 0.01

^*a*^Mean of triplicate results.

## Data Availability

The data used to support the findings of this study are included within the article.
